# In Vitro Antioxidant Activity Optimization of Nut Shell (*Carya illinoinensis*) by Extrusion Using Response Surface Methods

**DOI:** 10.3390/biom9120883

**Published:** 2019-12-16

**Authors:** Juliana Villasante, Esther Pérez-Carrillo, Erick Heredia-Olea, Isidoro Metón, María Pilar Almajano

**Affiliations:** 1 Chemical Engineering Department, Universitat Politècnica de Catalunya, Av. Diagonal 647, 08028 Barcelona, Spain; julianavillasante@gmail.com; 2 Centro de Biotecnologia FEMSA, Tecnologico de Monterrey, Av. Eugenio Garza Sada 2501, Monterrey, NL 64849, Mexico; perez.carrillo@tec.mx (E.P.-C.); erickho@tec.mx (E.H.-O.); 3 Biochemistry and Molecular Biology Section, Biochemistry and Physiology Department, Universitat de Barcelona, Joan XXII 27-31, 08028 Barcelona, Spain; imeton@ub.edu

**Keywords:** extrusion, pecan nut shell (*Carya illinoinensis)*, physicochemical properties, antioxidants, total dietary fiber

## Abstract

The pecan (*Carya illinoinensis*) nut shell is an important byproduct of the food processing industry that has not been previously explored as an antioxidant compound. This work aims to study the effect of the extrusion temperature and screw speed on the moisture content, water and oil absorption index, water solubility index, color, phenolic compounds, condensed tannin compounds, and antioxidant activity of pecan nut shell extrudates. Extrusion variables were adjusted using a response surface methodology. Extrusion, performed at 70 °C and 150 rpm, almost doubled the concentration of polyphenols in the non-extruded shell and significantly increased radical scavenging activity. Compounds in extrudates, performed at 70 °C and 150 rpm, were quantified by high-performance liquid chromatography (HPLC) with a diode-array detector (DAD) and identified by liquid chromatography coupled with time-of-flight mass spectrometry (LC-MSD-TOF). Extrusion significantly increased most phenolic acid compounds, including gallic acid, ellagic acid pentose, ellagic acid, dimethyl ellagic acid rhamnoside, and dimethyl ellagic acid. The soluble fiber in extrudates was more than three-fold higher than in the control. Therefore, extrusion at 70 °C and 150 rpm increased the concentration of phenolic compounds, antioxidant activity, and total dietary and soluble fiber. Our findings support the notion that extruded pecan nut shell can be used in clean-label products and improve their nutraceutical value.

## 1. Introduction

Pecan nuts (*Carya illinoinensis*) have been part of the human diet for hundreds of years and they are commonly used in the bakery and snack industry [1]. This type of nut has beneficial nutritional components, including polyunsaturated fatty acids, vitamins, minerals, protein, and other functional compounds, such as polyphenols [2]. Mexico and the United States are responsible for 93% of the world’s production of pecan nuts, with an average of nearly 60,000 and 40,000 metric tons per year, respectively [3]. However, between 40 and 50% of the original nut is wasted [4].

Prado et al. found that a pecan nutshell infusion presents antibacterial activity [5]. Müller et al. found that the shell infusion could be an economical agent in the prevention of liver diseases associated with ethanol consumption [6], additionally Reckziegel et al. found that the shell tea prevents the anxiety caused by cigarette abstinence, acting as a natural anxiolytic [7]. On the other hand, in the industry, this by-product could be used as a biosorbent or as precursor of carbon for the removal of dyes and heavy metals from aqueous solutions [8].

In recent years, consumers have demonstrated an interest in incrementing the consumption of foods with a high content of dietary fiber, with the idea to reduce the developing of chronical diseases [9]. The by-products of nut shells are composed of around 70–80% fiber that is predominantly insoluble fiber: lignin, cellulose, and hemicellulose [10]. In addition, they contain protein, various minerals, and also phenolic compounds and proanthocyanidins, including vanillic, caffeic, and gallic acids, catechin, and tannic acid, which could have an antioxidant effect on human and animal organisms [11,12,13].

Extrusion cooking has been commonly used for the development of new products, including snacks, breakfast cereals, and pet and baby foods. It is a technology that has a low cost, high productivity, versatility, and also improves the digestibility and nutritional bioavailability of the product for human and animal consumption [14]. Extrusion represents mechanical stress, which may alter physical and chemical characteristics [15]. It can be applied by controlling three independent variables, namely the barrel temperature, moisture, and screw speed [14,16,17]. The use of extrusion in the processing of by-products derived from cereals, fruits, and vegetables is of great interest. Several studies have focused on increasing the antioxidant activity and the polyphenols compounds and soluble fiber using this technology in wastes like soybean residue [18], shrimp shell [19], orange peel [20], bilberry press cake [21], and so forth. In addition to these improvements, extrusion has shown a benefit to the techno-functional characteristics of the byproducts. In the residues mentioned above, the water and oil absorption increased.

However, there have not been relevant reports about using twin-screw extruders in shells of nuts.

The aim of this work is to study the effects of extrusion process variables, including the barrel temperature and screw speed, on the chemical characteristics of pecan nut shells. The objective is to employ response surface methodology to optimize the extrusion process conditions on the total phenol content (TPC), condensed tannin contents (CTC), and antiradical activity (1,1-Diphenyl-2-Picrylhydrazyl; DPPH). The work includes the quantification of polyphenol compounds by HPLC, with a diode-array detector and the identification by LC-MSD-TOF (liquid chromatography time-of-flight mass spectrometry), and also the changes of fiber and protein contents in the samples under optimal experimental conditions (70 °C and 150 rpm).

## 2. Materials and Methods

### 2.1. Chemicals and Reagents

All the chemical reagents were obtained from Sigma-Aldrich (Sigma-Aldrich, St. Louis, MO, USA). The sunflower oil was from a local supermarket in Monterrey, Mexico. The total dietary fiber kit (K-TDFR-100A/) was purchased from Megazyme™ (Bray, Ireland).

### 2.2. Pecan Nut Shell Samples

The raw material used for non-extruded (control) and the extrusion process was pecan nut shell *(Carya illinoinensis)*. The pecan nut shells were separated manually from the kernel and collected at 1090 m above sea level (coordinates 25°45′32″ N, 102°58′58″ W) in San Pedro, Coahuila, which is located on the north of Mexico. The pecan nut shells collected were milled in a Wiley mill (Arthur Thomas, Philadelphia, PA, USA) equipped with a 2 mm screen.

### 2.3. Extrusion Processing

The shell powder was processed in a twin-screw co-rotating extruder (BCTM-30 Bühler, Uzwill, Switzerland) with an 800 mm length, 30 mm diameter, and L/D = 20 screws, with a configuration according to Cortés-Ceballos et al. [22] with some changes. The initial section comprised only conveying elements, the second included both conveying and mixing elements, and the last one, the shear section contained conveying, one reverse element and transport elements. A TT-137N water heater (Tool-temp, Sulgen, Switzerland) controlled the temperature at the final section of the extruding chamber. The shell was processed at a fixed feed rate of 7.79 kg/h dry matter. The extrudates and the control (non-extruded shell) were dried at 60 °C in an air convection oven (Edel Ingenieros, Monterrey, Mexico). Finally, all samples were stored at room temperature until analysis.

### 2.4. Experimental Design and Extrusion Conditions

A face-centered central composite RSM (Response Surface Methodology) was used to determine the effects of extrusion on total phenols, condensed tannins and DPPH radical scavenging activity. Two different factors were evaluated: screw speed and barrel temperature in the range: temperature (minimum = 33.25 °C, maximum = 106.75 °C) and screw speed (minimum = 88.75 rpm, maximum = 211.75 rpm). The experimental design to obtain extruded nut shells with two factors is found in Table 1.

### 2.5. Techno-Functional Characterization

The moisture of the extrudates and control (non-extruded shell) samples was determined by the method of AOAC 925.10 [23] just after the extrusion process. The color measurements of shell extrudates and the control (non-extruded shell) were performed using a Konica Minolta CM-600d colorimeter, calibrated with a standard series (𝑋 = 94.9, 𝑦 = 0.32, and 𝑥 = 0.31). The luminosity was determined by L*, red-green by a*, and yellow-blue by b*. Parameters a* and b* were used to calculate chrome (C*) and Hue angle (ℎ °) according to Equations (1) and (2):

(1) $$C^{*}\;=\;\sqrt{a^{*2}+b^{*2}},$$

(2) $$h{}^{\circ}\;=\;\tan^{-1}\;(b^{*2}/a^{*2}).$$

The water absorption index (WAI) was determined according to the method described by Ruiz-Gutiérrez et al. [24]. The extruded shell or the control (non-extruded shell) (2.5 ± 0.05 g) was suspended in distilled water (30 mL) at 25 °C and then centrifuged at 3000× *g* for 10 min. The supernatant was removed. The WAI was calculated as the weight of sediment per weight of dry solid shell. The water solubility index (WSI) was the weight of dry solids in the supernatant from the WAI test after decantation and evaporation at 105 °C. The oil absorption index (OAI) was determined by the Ruiz-Gutie Rui et al. [24] method with few modifications. The extruded shell and the control (non-extruded shell) (2.5 ± 0.05 g) was suspended in sunflower oil (10 mL) at 25 °C, then stirred for 30 s, and then centrifuged at 3000× *g* for 10 min. The supernatant was removed. The OAI was calculated as the weight of sediment per weight of dry solid shell.

### 2.6. Phenolic Determination

#### 2.6.1. Extraction for Total Phenolic Compounds 

Dried extruded shells and the control (non-extruded shell) were weighed (1 ± 0.05 g) and extracted with 20 mL of an ethanol-water mixture at 50:50 (*v*/*v*). The mixture was stirred for 90 min at 25 °C. All samples were centrifuged (Sigma 6K10, Osterode am Harz, Germany) for 15 min at 3000× *g*.

The Folin–Ciocalteu method was used to determine the TPC, as reported by Singleton et al. [25], using a ultraviolet (UV)-Vis microplate reader spectrophotometer Fluostar Omega (Paris, France) at 25 °C; the results were expressed in mg gallic acid equivalents/g dry weight (mg GAE/g DW). The standard curve was obtained by plotting the absorbance against different concentrations of gallic acid (ranging from 0.12 to 1.73 mM).

#### 2.6.2. Condensed Tannin Content 

The vanillin assay method, according to Flores-Córdova et al. [26], was followed with some modifications to quantify the CTC. Hereby, 50 µL of the shell extract (1:20 (*w*/*v*) in ethanol-water at 50:50 (*v*/*v*)) was added to 4 mL of 37% HCl solution with 8% MeOH (*v*/*v*) and a vanillin solution prepared in 4% MeOH (*v*/*v*) at a proportion of 50:50 (*v*/*v*).

Following 20 min of incubation in the dark, the solution was measured at 500 nm in a UV-Vis microplate reader spectrophotometer Fluostar Omega (Paris, France). The CTC was expressed as mg equivalents of catechin/dry weight of the sample (mg EC/g DW). The standard curve was obtained by plotting the absorbance against different concentrations of catechin (ranging from 0.3 to 1.5 mg/mL).

#### 2.6.3. Determination of 1,1-Diphenyl-2-Picrylhydrazyl Radical Scavenging Activity

The DPPH free radical scavenging activity of the control (non-extruded shell) and extruded shell treatments was measured according to the method described by Villasante et al. [27]. An initial absorbance measurement of the DPPH reagent was recorded (*A*_0_). The shell extracts (same extraction used for TPC 2.6.1.) in five different dilutions reacted with 200 μL of DPPH in methanol for a short period of time. The absorbance (*A*_1_) was measured at 517 nm after a period of 75 min using a UV-Vis microplate reader spectrophotometer Fluostar Omega (Paris, France) at 37 °C. The inhibition percentage of radical scavenging activity of each treatment was calculated by the following equation (Equation (3)).

(3) $$\%\;inhibition\;of\;sample=\left(\frac{A{\;}_{\;0}-\;A{\;}_{\;1}}{A{\;}_{0\;}}\right)\times 100,$$

where A_0_ is the absorbance of DPPH without sample and A_1_ is the absorbance of the extracts at time 75 min. All analyses were conducted in triplicate. The results are presented as means ± standard deviation (SD) based on the three field replicate samples. Half inhibitory concentration (IC_50_) of DPPH radical was calculated based on the liner regression of the percentage of the remaining DPPH radical against the sample concentration.

### 2.7. Optimized Treatment Characterization

An optimum treatment was produced by applying the regression obtained from the RSM in which the DPPH capacity was maximized. This treatment was characterized.

#### 2.7.1. Free Phenolic Compounds Extraction and Identification and Quantification

From optimized treatment, FPC (free phenolic compounds) were extracted using the method described by Adom and Liu [28] with some modifications. One g of the extruded shell and the control (non-extruded shell) were mixed with 10 mL of ethanol-water 50:50 (*v*/*v*) and vortexed for 10 min. The mixture was centrifuged for 10 min at 3000× *g*. The ethanol was evaporated with nitrogen at room temperature, then the extracts were lyophilized and stored at −20 °C in the darkness until analysis. The phenolic compounds in control and optimized treatment were identified and quantified according to Acosta-Estrada et al. [29], with few modifications. The lyophilized samples were dissolved in 1 mL of methanol and then filtered through 0.45 µm nylon filter. The compounds were quantified by high-performance liquid chromatography with a diode-array detector (HPLC–DAD) (1200 Series, Agilent Technologies, Santa Clara, CA, USA) using a reverse-phase column (Zorbax SB-Aq, Santa Clara, CA, USA) 4.6 mm ID × 150 mm (3.5 µm) scanning at different wavelengths of 254, 280, and 320 (obtained from an sample scanning). Chromatographic separations were performed using a mixed mobile phase composed of (A): water acidified (pH = 2) with formic acid and (B): acetonitrile. The flow rate was 0.6 mL/min at 25 °C. The gradient was as follows: 0–10 min 15% B, 10–14 min 58% B, 14–20 min 80% B, and 20–30 min 100% B. Identification was performed using liquid chromatography coupled to time-of-flight mass spectrometry (LC-MSD-TOF) (1100 Series, Agilent Technologies, Santa Clara, CA, USA) under the same chromatographic conditions described for the HPLC-DAD analysis, with an electrospray source in positive ion mode (ESI+) with the following parameters: nitrogen gas temperature, 300 °C; drying gas flow rate, 8 L/min; curtain gas, 50 psig; capillary voltage, 4000 V; and fragment voltage, 70 V. MS spectra were recorded in the range of *m/z* 100–1000. The quantification of phenolic compounds, for which standards were available, was carried out using appropriate calibration curves. Spectral data was collected using Mass Hunter (Santa Clara, CA, USA, (A.02.01(B730)), Agilent Technologies, CA, USA) workstation software.

#### 2.7.2. Protein and Dietary Fiber Determination

The crude protein content was determined by the Kjeldahl digestion and distillation methods (No. 920.87 AOAC) [23]. A conversion factor of 6.25 was selected to calculate the protein content. The in vitro protein digestibility assay was used for analyzing the digestibility of protein samples [30]. The analysis was carried out by a multi-enzyme solution: trypsin, chymotrypsin, and peptidase, that was added to the solution of the sample with pH = 8. The sample and enzyme solution was mixed and stirred for 10 min at 37 °C. The resulting pH was recorded for 10 min and the in vitro protein digestibility was calculated with Equation (4) (where the ΔpH_10min_ is the change in pH in 10 min from the initial pH of 8.0):

*IVDP*% = 65.66 + 18.10·ΔpH_10min_. (4)

The standardized enzymatic-gravimetric method (AOAC 991.43) was used for the determination of insoluble (IDF) and soluble dietary fiber (SDF) content. Total dietary fiber (TDF) was calculated as the sum of SDF and IDF [9].

### 2.8. Statistical Analysis

For the techno-functional characterization, each experiment was performed in triplicate and data were reported as means ± standard deviations. Results were subjected to analysis of variance with ANOVA and differences among means were compared by the Tukey test (*p* < 0.05). Statistical analysis was performed with software MINITAB 18^®^-0 (Minitab Inc., State College; PA, USA).

The RSM data was submitted to the MINITAB 18^®^-0 and fitted to a second-order polynomial model, and regression coefficients were obtained with a significance level (α) of 0.05. The generalized second-order polynomial model used in the response surface analysis followed the Equation (5)

(5) $$Y=\beta_{0}+\;\sum_{i}^{j}\beta_{i}X_{i}+\;\sum_{i}^{j}\beta_{ii}X_{i}^{2}\;+\;\sum_{i}^{j}\beta_{ij}X_{i}X_{j},$$

where $\beta_{0}$ is the model constant coefficient, $\beta_{i}$ is the linear coefficients, $\beta_{ii}$ is the quadratic coefficient and $\beta_{ij}$ is the interaction coefficient of variables *i* and *j*.

The statistical analysis for the optimized treatment characterization was the same as that used for the techno-functional characterization.

## 3. Results and Discussion

### 3.1. Physical Characterization

Although in the extrusion process the water content remained constant, the moisture content of the extruded shell was significantly affected (*p* < 0.05) by the extrusion treatment (Table 2). Compared to control samples (non-extruded shell), extrusion significantly decreased the moisture content 1.2- to 1.4-fold. The screw speed and the temperature affected the moisture percentage. This difference in moisture content could be explained by the effect of the temperature and the speed screw on the material structure. In a study presented by Borchani et al. it has been found that in a by-product from dates with the incorporation of a temperature treatment, the dry matter increased as well as the content of TDF [31]. Several authors suggest that products with high dry matter contents contribute to an easier auto conservation of fiber [32,33].

In the case of WAI and OAI, only samples T2 and T7 were significantly higher than control values (Table 2). However, in the case of WSI, most of the samples were significantly different than the control. Condition T11 (106.75 °C, 150 rpm) produced the highest WSI (%) value: 4.47 ± 0.10 versus 3.00 ± 0.14 in the control (non-extruded shell). Consistently, similar results were obtained in agro-industrial by-products extrudates like rice bran and paddy husk, the WSI increased with the increment of temperature up to 110 °C [34]. Extruders will provide high shear, rapid heat transfer, and effective mixing in a short residence time. The physical and chemical structure of the material will be disturbed and changed during the passage through the extruder barrel, resulting in a large specific area to increase. Jan et al. and Zheng and Rehmann support the results with the thermal degradation during the extrusion process (high temperatures), as this could be the reason for the physical and chemical change on the material structure, resulting in an increment of the accessibility of cellulose for enzymatic action [34,35].

The results in luminosity (L*), red-green (a*) and yellow-blue (b*) parameters, the chroma (C) and hue angle (ℎ∘) of shell extrudates, and the control (non-extruded shell) are presented in Table 2. According to the results, the extrusion conditions evaluated had an important effect on color parameters. Significant difference was detected when comparing the L* value of the control (non-extruded) and all the extrusion treatments, within the range of 10.92–23.37, respectively. Similar results were obtained with carrot pomace-based extrudates, where the increase of the L value was observed with the increment of temperature. This effect shows the relation with the increase in brightness and rise in air cells [36].

The screw speed and the temperature also had a significant effect on the a* and b*. The maximum a* (10.58) and b* (14.63) values were reported with treatment 11 (150 rpm, 106.75 °C) while a minimum a* (6.51) and b* (7.88) values were revealed for the control. Increase of red-green and yellow-blue could be related to the varied structure due to higher extruder temperature. A maximum change was produced at a screw speed of 150 and temperature of 106.75 °C on red-green and yellow-blue in comparison with the control: a* (10.58–6.51) and b* (14.63–7.88). Likewise, relating the chroma and hue angle increased the values with the extrusion process. Regarding C* and ℎ∘, non-extruded samples showed the lowest values 10.23 and 50.39 respectively. Treatment 11 (150 rpm and 106 °C) showed the higher value for C* with 18.06 and for ℎ∘ the treatment 9 (100 rpm and 100 °C) with a value of 54.17.

The values of the extrudates samples (L*, C*, ℎ∘) may be affected by the destruction of thermosensitive pigments. Jan et al. [34,37] observed similar results in agro-industrial wastes, and the authors found that the extruder variables had an effect on the color. Thermal processing of food produces reactions, such as Maillard, caramelization, or hydrolysis, which can affect the color in extruded products. Melanoidins are represented by dark color pigments [38,39,40]. Until now, the effect of the extrusion process on the color of fibrous materials, such as the shell, has not been researched.

### 3.2. Chemical Characterization

Table S1 (Total phenolic contents (TPC), condensed tannins, and DPPH assay (IC_50_) after 50% ethanol extraction) and Table S2 (Coefficients of extrusion conditions variables (B temperature and A screw speed) of the predictive quadratic model for Total Phenolic Content (TPC), Radical Scavenging Activity (DPPH) and Condensed Tannin Contents (CTC)) are in the Supplementary Materials.

Estimated regression coefficients for TPC, DPPH assay (IC_50_), and CTC were expressed as coded units in second order polynomial equations (Equations (6)–(8)) as follows (A: screw speed (rpm); B: temperature (°C)):

(6) $$TPC\;\left(\mathrm{mg}\frac{GAE}{gDW}\right)=-185.2\;+\;1.780\;A\;+\;3.187\;B-\;0.005900\;A*A\;–\;0.02210\;B*B\;$$

(7) $$CTC\;\left(\mathrm{mg}\frac{GAE}{gDW}\right)=208.0\;–\;0.425\;B\;+\;3.238\;A\;+\;0.00236\;B*B\;–\;0.00540\;A*B\;$$

(8) $$DPPH\;\left(IC50\right)\left(µg\frac{DW}{ml}\right)=\;2547\;–15.74\;B\;–25.25\;A\;+\;0.0491\;B*B\;+\;0.1651\;A*A.$$

#### 3.2.1. Total Phenolic Content 

**Figure 1:**
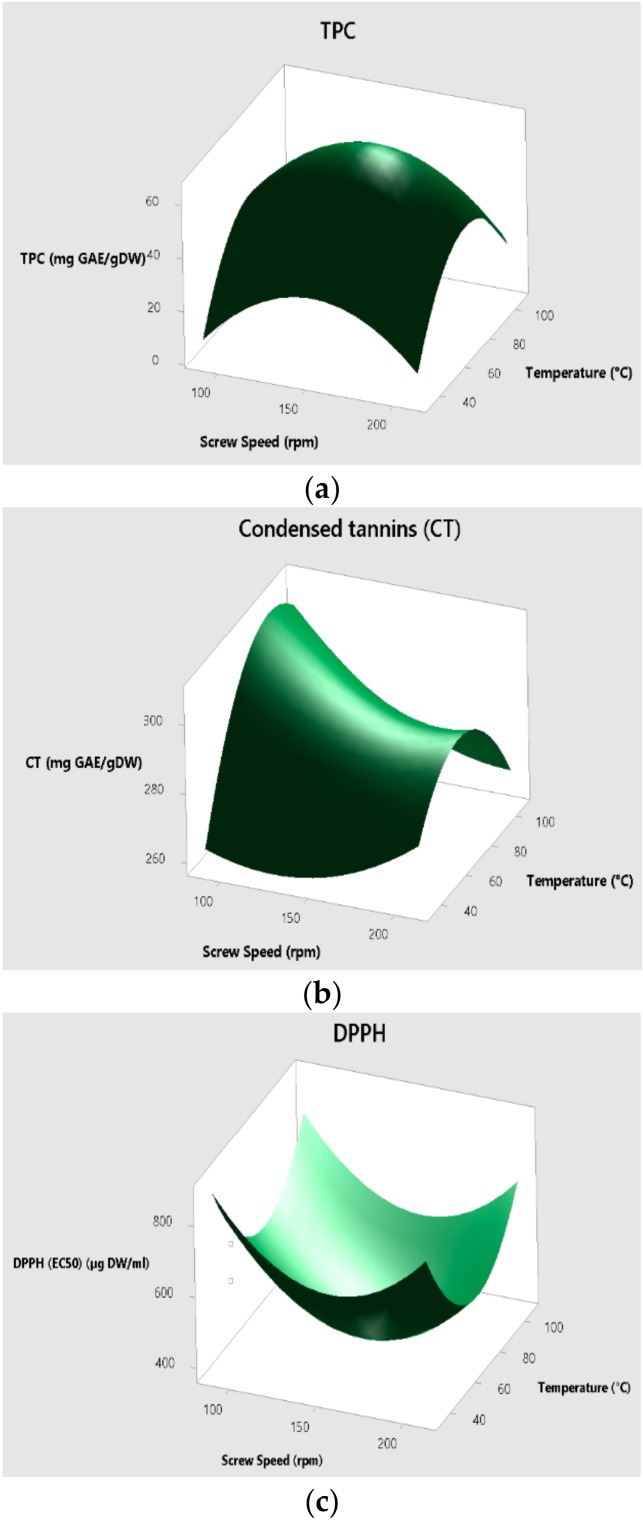
Reponses surface for total phenolic content (TPC) (**a**), condensed tannins (**b**), and DPPH (1,1-Diphenyl1-2-pircrylhydrazyl) (**c**) in pecan nut shell extruded at different temperatures (33.25 °C, 40 °C, 70 °C, 100 °C, or 106.5 °C) and velocities (88.75, 100, 150, 200, and 211.25 rpm).

The coefficient of determination (R^2^) of the model was 0.8854, which indicated that 11.46% of the total variation was not explained by the model. A second order polynomial equation (Equation (6)) was obtained by depicting the correlation between the temperature and screw speed with TPC. A negative quadratic effect of the screw speed and the temperature exits. Figure 1a shows that the temperature, screw speed, and the interaction between both of them show no significant variables in the process. The smaller the *p*-value was, the more significant the corresponding coefficient was [18].

Previous studies showed that the extrusion-cooking process increased the phenolic content and the antioxidant activity. Brennan et al. [14] assumed that increased levels of certain phenolic components in extruded products are frequently due to their release from the cell wall matrix. However, in a study by Mahungu et al. [41], it was found that the barrel temperature in the extrusion process plays a significant role in the stability of phenolic compounds, such as isoflavones in soy products. The increase in phenolic content with barrel temperature has been ascribed to the formation of Maillard reaction products [42]. The effect of extrusion on bioactive compounds depends on the physical and chemical characteristics of the product and the extrusion conditions [43,44].

#### 3.2.2. Condensed Tannin Contents 

Pecan nut shell contains high concentrations of condensed tannins that make pecans an interesting source of phytochemicals [13]. Similar results reported by Adarkwah-Yiadom and Duodu [45] showed that the extrusion cooking of whole grain sorghum (type II and type III) significantly reduced condensed tannins (Figure 1b). The authors concluded that this might be due to tannin interaction with protein and other macromolecules, which reduces their extractability [45]. Unlike the TPC, the CTC has a negative correlation with the temperature and the interaction between speed screw and temperature (Equation (7)). A positive correlation was presented by the quadratic of the temperature.

The temperature (*p*-value 0.024) and the interactions between temperature–screw speed (*p*-value 0.021) were statistically significant, but the screw speed was not significant (*p*-value 0.064).

#### 3.2.3. Radical Scavenging Activity 

There are some variables to consider in order to improve the recovery of polyphenols and antioxidants [25]. The radical scavenging activity (RSA) of extruded products depends not only on the phenolic compounds but also on the interactions between these bioactive compounds and the food matrix [46]. The antioxidant capacity of extrudates and non-extruded control samples was determined using the DPPH assay, a sensitive electron-transfer reaction [47]. Figure 1c shows that the highest RSA (lowest IC_50_) was obtained for samples processed at 70 °C and 150 rpm. The second order polynomial equation (Equation (8)) was obtained by depicting the correlation between the temperature and screw speed with the DPPH assay (IC_50_). The modeling was fitted as a linear regression (*R*^2^ 87.06%).

The extrusion process increased antioxidant activity in most of the treatments. The same tendency was previously observed by Ramos-Enríquez et al. [48], who, by means of RSM showed that high temperatures increased the RSA of wheat bran. However, it was shown that the antioxidant activity of the lentil-orange (raw sample) was reduced from 95.6 to 60.6% by extrusion [49].

### 3.3. Polyphenolic Compounds, Dietary Fiber, and Protein Composition

The identification and quantification of polyphenolic compounds and total dietary fiber and protein contents were determined for samples obtained with the treatment that produced the highest amount of total polyphenols and the lowest IC_50_ (the highest radical scavenging activity (DPPH)): 70 °C and 150 rpm.

#### 3.3.1. Identification and Quantification of Polyphenolic Compounds by LC-MSD-TOF

**Figure 2:**
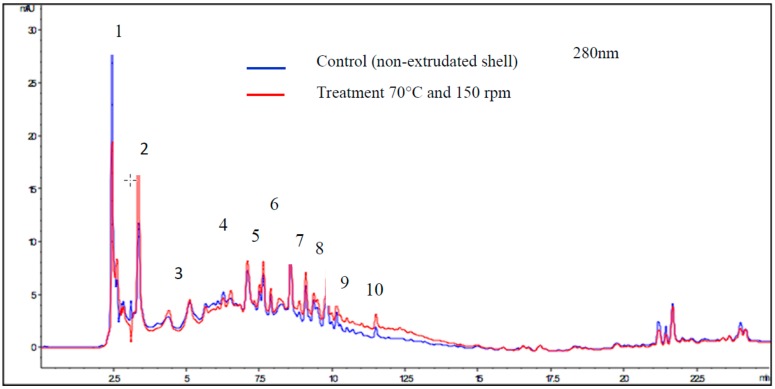
HPLC-DAD (high pressure liquid chromatography-diode-array detector) chromatograms of compounds in control (non-extruded shell) and milled shells extruded at 70 °C and 150 rpm with detection at 280 nm. Blue: control. Red: treatment 70 °C and 150 rpm. Retention time (t_R_, min).

Table 3 shows the parameters of identification and quantification of each phenolic compound obtained for the control (non-extruded shell) and the extruded shell after treatment at 70 °C and 150 rpm. The samples contained several phenolic acids including gallic, ellagic, p-hydroxybenzoic, protocatechuic, and also pentose, methyl ellagic pentoside, epigallocatechin gallate, dimethyl ellagic rhamnoside, and dimethyl ellagic acid. Figure 2 shows an HPLC chromatogram measured at 280 nm. For both the control and treatment (70 °C and 150 rpm) samples, the major phenolic acid compound was ellagic acid (1.56 and 1.74 μg/g DS, respectively). However, in the control extract (non-extruded shell), dimethyl ellagic acid rhamnoside was not identified. After the treatment, the samples presented significantly higher concentrations of phenolic compounds than the control (non-extruded shell): gallic acid, ellagic acid pentose, ellagic acid, dimethyl ellagic acid rhamnoside, and dimethyl ellagic acid.

A study by Hilbig et al., which evaluated the effects of different conditions involving ultrasound extraction of the total phenolic content from pecan nut shells, found that catechin was the most concentrated compound in shell extracts, followed by gallic acid [50]. The concentrations reported by the authors [50] were higher than those observed in the present study (all the treatments). This could be attributed to the extraction process and the origin of the nuts. Moreover, Gulati et al. found that *Panicum miliaceum* L. flour roasted for 10 min at 110 °C increased the content of secondary compounds and the antioxidant properties. HPLC manifested a higher amount of ferulic acid, among other acids [51].

#### 3.3.2. Protein and Fiber Content

High temperature and pressure may alter protein structure, leading to protein denaturation and the formation of cross-linking reactions [52]. Therefore, it is likely that in the present study, pressure and temperature affected protein structure during the extrusion process, decreasing protein extractability. In fact, the protein content in the extruded sample was lower than in the control (non-extruded shell). Although, the in vitro digestibility of protein (IVDP%) increased significantly with the extrusion process (Table 4).

Dietary fiber are usually divided into SDF and IDF. SDF is composed of pectin and gums, and IDF by cellulose and lignin [53]. SDF has an important role in the food industry thanks to its beneficial physiological functions in the organism [53]. Table 4 shows the content of the total soluble and insoluble dietary fiber in non-extruded and extruded shell at 70 °C and 150 rpm. TDF in the extruded sample was significantly higher than in the control levels, 75.41 and 79.10, respectively. Likewise, under the same extrusion conditions (70 °C and 150 rpm), SDF was significantly higher than in the control. A recent study reported by Ge et al. [54] showed that the extrusion process applied to bamboo shoot flours caused a similar tendency, and the results led to conclude that the process of extrusion may break glycosidic linkages in TDF, causing the conversion of IDF into SDF. Another study with banana peel reported that temperature and screw speed conditions in extrusion process can enhance transformation of IDF to SDF [55]. This could result from the release of oligosaccharides and polysaccharides (cellulose, hemicellulose, and lignin). Similar results are presented in a work about the extrusion of soybean residues, they found that with extrusion temperature of 115 °C; feed moisture, 31%; and screw speed, 180 rpm, the SDF content of soybean residue increased 10.60% compared with the un-extruded soybean residue [18].

In this regard, Hu el al. reported that samples with higher soluble fiber have greater water and oil absorption [16]. We observed that extrusion produced, in general, higher concentrations of SDF, which were associated with an increased solubility in water [56]. Table 2 shows that the treatments with 150 rpm and 70 °C (4, 6 and 8) present similar results of WAI, OAI, and WSI. Furthermore, there exists a significant increment of the WAI and WSI values with respect to the control.

## 4. Conclusions

This research uses a response surface method to determine the effect of extrusion techno-functional properties and antioxidant activity. The extrusion process affected significantly the techno-functional properties of pecan nut shell, such as the moisture and color. When extrusion was performed at 70 °C and 150 rpm, the extruded nut shell contained more total polyphenols and soluble dietary fiber and presented higher radical scavenging activity in comparison to the non-extruded nut shell control. In summary, these results demonstrate that twin-screw extrusion could be used as a tool to generate novel food ingredients with modified functionality, using industry wastes as raw materials. Moreover, the nut shell could be used in food formulations as both a fiber ingredient and additive, due to its antioxidant capacity.

## Figures and Tables

**Table 1:** Experimental design to obtain extruded nut shells with two factors.

Treatment	Order Run	Replicates	Factors	
			Temperature (°C)	Screw Speed (rpm)
1	2	2	33.25	150
2	11	2	40	100
3	4	2	40	200
4	8	2	70	150
5	10	2	70	88.75
6	9	2	70	150
7	7	2	70	211.25
8	1	2	70	150
9	3	2	100	100
10	6	2	100	200
11	5	2	106.75	150

**Table 2:** Moisture, color, and techno-functional properties of pecan nut shell without extrusion and extruded at different temperatures (33.25, 40, 70, 100, or 106.5 °C) and velocities (88.75, 100, 150, 200, and 211.25 rpm).

Treatment	Moisture (%)	WAI (g)	OAI (g)	WSI (%)	L*	a*	b*	C* (Chroma)	ℎ∘ (Hue Angle)
Control	5.97 ± 0.06 ^a^	1.96 ± 0.03 ^c^	2.40 ± 0.02 ^cde^	3.00 ± 0.14 ^f^	10.92 ± 0.68 ^g^	6.51 ± 0.42 ^f^	7.88 ± 0.75 ^g^	10.23 ± 0.47 ^f^	50.39 ± 0.00 ^b^
T1 (33.25 °C, 150 rpm)	4.74 ± 0.09 ^bc^	2.88 ± 0.10 ^abc^	2.29 ± 0.01 ^e^	3.70 ± 0.03 ^de^	14.83 ± 0.05 ^f^	8.81 ± 0.22 ^de^	11.93 ± 0.07 ^f^	14.83 ± 0.06 ^de^	53.60 ± 0.68 ^a^
T2 (40 °C, 100 rpm)	4.21 ± 0.04 ^d^	3.33 ± 0.10 ^ab^	3.02 ± 0.01 ^a^	3.44 ± 0.01 ^e^	14.40 ± 0.09 ^f^	9.95 ± 0.01 ^b^	13.73 ± 0.15 ^b^	16.96 ± 0.00 ^b^	54.10 ± 0.05 ^a^
T3 (40 °C, 200 rpm)	4.88 ± 0.05 ^b^	2.26 ± 0.08 ^c^	2.54 ± 0.01 ^bc^	4.360 ± 0.09 ^b^	22.42 ± 0.08 ^b^	10.74 ± 0.06 ^a^	14.49 ± 0.02 ^a^	18.04 ± 0.09 ^a^	54.15 ± 0.41 ^a^
T4 (70 °C, 150 rpm)	4.40 ± 0.06 ^cd^	2.55 ± 0.31 ^abc^	2.42 ± 0.01 ^cde^	3.50 ± 0.01 ^e^	16.66 ± 0.03 ^e^	9.23 ± 0.14 ^c^	12.24 ± 0.14 ^ef^	15.33 ± 0.02 ^cd^	53.01 ± 0.05 ^ab^
T5 (70 °C, 88.75 rpm)	4.68 ± 0.06 ^bc^	2.43 ± 0.22 ^bc^	2.37 ± 0.02 ^cde^	4.36 ± 0.06 ^b^	19.64 ± 0.03 ^c^	10.13 ± 0.01 ^b^	13.26 ± 0.01 ^bc^	16.68 ± 0.02 ^b^	52.64 ± 0.03 ^ab^
T6 (70 °C, 150 rpm)	4.39 ± 0.02 ^cd^	2.55 ± 0.32 ^abc^	2.39 ± 0.01 ^cde^	3.59 ± 0.14 ^e^	16.22 ± 0.10 ^e^	9.19 ± 0.09 ^cd^	12.38 ± 0.15 ^ef^	15.41 ± 0.15 ^c^	53.41 ± 0.05 ^ab^
T7 (70 °C, 211.25 rpm)	4.60 ± 0.01 ^bc^	3.55 ± 0.67 ^a^	2.74 ± 0.00 ^b^	4.14 ± 0.06 ^bc^	19.55 ± 0.00 ^c^	10.02 ± 0.01 ^b^	13.06 ± 0.06 ^cd^	16.46 ± 0.00 ^b^	52.52 ± 0.03 ^ab^
T8 (70 °C, 150 rpm)	4.42 ± 0.09 ^cd^	2.50 ± 0.24 ^bc^	2.27 ± 0.18 ^e^	3.58 ± 0.15 ^e^	16.38 ± 0.12 ^e^	9.19 ± 0.04 ^cd^	12.46 ± 0.21 ^def^	15.48 ± 0.05 ^c^	53.59 ± 0.03 ^a^
T9 (100 °C, 100 rpm)	4.15 ± 0.14 ^d^	2.56 ± 0.41 ^abc^	2.33 ± 0.01 ^cde^	3.53 ± 0.01 ^cd^	16.66 ± 0.14 ^e^	8.66 ± 0.15 ^e^	11.90 ± 0.01 ^f^	15.33 ± 0.02 ^e^	54.17 ± 0.05 ^a^
T10 (100 °C, 200 rpm)	4.95 ± 0.01 ^b^	2.43 ± 0.22 ^bc^	2.31 ± 0.01 ^de^	2.87 ± 0.14 ^f^	17.78 ± 0.14 ^d^	9.27 ± 0.14 ^c^	12.69 ± 0.01 ^cde^	15.72 ± 0.01 ^c^	53.86 ± 0.07 ^a^
T11 (106.75 °C, 150 rpm)	4.59 ± 0.02 ^bc^	2.42 ± 0.22 ^bc^	2.52 ± 0.01 ^cd^	4.47 ± 0.10 ^a^	23.37 ± 0.01 ^a^	10.58 ± 0.02 ^a^	14.63 ± 0.03 ^a^	18.06 ± 0.01 ^a^	53.48 ± 0.02 ^ab^

Average ± standard deviation (SD) (*n* = 3); different letters by column represent significant differences by Tukey’s test (*p* < 0.05). WAI: Water absorption index; OAI: Oil absorption index; WSI: Water solubility index.

**Table 3:** Phenolic profile (μg/g dry shell) of pecan nut shell extrudate ethanolic extracts, with HPLC-DAD and LC-MSD-TOF, detected at 280 nm.

Peak	UV/MAX	Phenolic Compounds	[M − H]^−^ *m/z*		Molecular Weight Da	t_R_ (min)	Content in Control (Non-Extruded Shell) (μg/g DS)	Content in 70 °C and 150 rpm (μg/g DS)
			Experimental Mass (*m/z*)	Theoretical Mass (*m/z*)				
1	215, 270	p-hydroxybenzoic acid	137.01	137.02	138.12	2.49	1.46 ± 0.17 ^a^	0.88 ± 0.10 ^b^
2	213, 271	Gallic acid	169. 02	169.08	170.12	3.35	0.90 ± 0.08 ^a^	1.55 ± 0.09 ^b^
3	231, 259	Protocatechuic acid	153.02	153.02	154.12	5.11	NQ	NQ
4	252, 360	Ellagic acid pentose	433.05	433.05	434.31	7.87	0.32 ± 0.06 ^a^	0.41 ± 0.09 ^b^
5	255, 368	Ellagic acid	301.01	301.00	302.19	8.56	1.56 ± 0.23 ^a^	1.74 ± 0.17 ^b^
6	254, 286	Methyl ellagic acid pentoside	477.08	477.08	448.33	9.06	NQ	NQ
7	223, 289	Epigallocatechin gallate	457.18	457.18	458.37	9.74	NQ	NQ
8	221, 251, 365	Di-methyl ellagic acid rhamnoside	475.10	475.10	476.39	10.13	NQ	0.32 ± 0.02 ^a^
9		Di methyl ellagic acid	329.04	329.04	330.25	10.68	0.32 ± 0.05 ^a^	0.33 ± 0.04 ^a^

Average ± SD; different letters by column represents significant differences by Tukey’s test (*p* < 0.05). NQ—the samples were detected but not quantified because the levels were under the quantification limit.

**Table 4:** Protein and dietary fiber content in the control (non-extrudated pecan nut shell) and optimized extrudate of pecan nut shell.

Treatment	Protein %	IVDP %	TDF %	SDF %	IDF %
Control (non-extruded)	2.56 ± 0.25 ^a^	85.07 ± 0.11^b^	75.41 ^b^	0 ± 0 ^b^	75.41 ± 1.7 ^a^
Optimized extruded (70 °C and 150 rpm)	2.41 ± 0.21 ^b^	87.70 ± 0.20^a^	79.1 ^a^	3.07 ± 0.21 ^a^	76.03 ± 1.59 ^b^

Average ± standard deviation; different letters by rows represent significant differences by Tukey’s test (*p* < 0.05).

## Supplementary Materials

The following are available online at https://www.mdpi.com/2218-273X/9/12/883/s1, Table S1: Total phenolic contents (TPC), condensed tannins, and DPPH assay (IC_50_) after 50% ethanol extraction; Table S2: Coefficients of extrusion conditions variables (B temperature and A screw speed) of the predictive quadratic model for Total Phenolic Content (TPC), Radical Scavenging Activity (DPPH) and Condensed Tannin Contents (CTC).
