# Supplementary material for: In Vitro Antioxidant Activity Optimization of Nut Shell (Carya illinoinensis) by Extrusion Using Response Surface Methods
Source: Biomolecules. 2019 Dec 16;9(12):883. doi: 10.3390/biom9120883 (PMC6995571; doi:10.3390/biom9120883)
Supplement: Supplementary file 1 [file biomolecules-09-00883-s001.pdf]

Table S1. Total phenolic contents (TPC), condensed tannins, and DPPH assay (IC_{50}) after 50 % ethanol extraction.

Treatment	TPC (mg GAE/gDW)	Condensed Tannins (mg GAE/gDW)	DPPH (IC_{50}) (μg DW/ml)
Control (non-extruded shell)	36.14±0.09[e]	309.60±0.22[a]	617.28±0.33[cd]
T1 (33.25°C, 150 rpm)	21.29±0.01[h]	262.11±0.84[h]	749.76±1.44[a]
T2 (40°C, 100 rpm)	32.82±0.02[g]	264.61±0.14[g]	668.11±2.46[b]
T3 (40°C, 200 rpm)	34.98±0.02[f]	284.30±0.05[e]	614.16±6.27[cd]
T4 (70°C, 150 rpm)	69.83±0.05[a]	291.56±0.49[c]	377.54±1.41[f]
T5 (70°C, 88.75 rpm)	36.40±0.03[de]	310.56±0.35[a]	681.46±2.34[b]
T6 (70°C, 150 rpm)	65.21±0.50[c]	287.40±0.07[d]	368.94±2.29[f]
T7 (70°C, 211.25 rpm)	37.33±0.03[d]	277.53±0.49[f]	568.34±1.77[e]
T8 (70°C, 150 rpm)	67.08±0.21[b]	293.15±0.23[c]	381.76±7.85[f]
T9 (100°C, 100 rpm)	34.48±0.03[f]	299.92±0.19[b]	623.03±1.75[c]
T10 (100°C, 200 rpm)	37.34±0.03[d]	287.20±0.23[d]	603.70±2.76[d]
T11 (106.75°C,150 rpm)	37.04±0.04[de]	265.23±0.75[g]	577.47±2.15[e]

Data from control and extrusion treatments; all contents are expressed *versus* dry plant material. Average ± SD; different letters by column represents significant differences by Tukey's test ($p < 0.05$).

Table T2. Coefficients of extrusion conditions variables (B temperature and A screw speed) of the predictive quadratic model for Total Phenolic Content (TPC), Radical Scavenging Activity (DPPH) and Condensed Tannin Contents (CTC).

Factor	TPC				DPPH				CTC			
	**Coef**	**Std Error**	***T*-Value**	***p*-Value**	**Coef**	**Std Error**	***T*-Value**	***p*-Value**	**Coef**	**Std Error**	***T*-Value**	***p*-Value**
Linear coefficient	65.03	2.43	26.81	0.000	392.2	21.1	18.63	0.000	289.32	3.58	80.88	0.000
A (rpm)	1.08	2.00	0.54	0.596	-37.2	17.3	-2.15	0.048	−585	2.94	−1.99	0.064
B (°C)	4.08	2.00	2.04	0.058	-46.6	17.3	-2.69	0.016	7.35	2.94	2.50	0.024
A(rpm)*A (rpm)	-22.13	3.24	−6.84	0.000	184.3	28.1	6.56	0.000	8.85	4.77	1.85	0.082
B (°C)*B (°C)	-29.84	3.24	−9.22	0.000	223.1	28.1	7.94	0.000	−21.49	4.77	−4.50	0.000
A (rpm)*B (°C)	0.26	3.23	0.08	0.937	12.9	28.1	0.46	0.653	−12.16	4.77	−2.55	0.021
R^2	88.54				87.06				72.70			
R^2 adj.	84.96				83.01				64.17			
